# Nonlinear observer output-feedback MPC treatment scheduling for HIV

**DOI:** 10.1186/1475-925X-10-40

**Published:** 2011-05-27

**Authors:** Ryan Zurakowski

**Affiliations:** 1 Department of Electrical and Computer Engineering, University of Delaware, Newark, DE 19716, USA

## Abstract

**Background:**

Mathematical models of the immune response to the Human Immunodeficiency Virus demonstrate the potential for dynamic schedules of Highly Active Anti-Retroviral Therapy to enhance Cytotoxic Lymphocyte-mediated control of HIV infection.

**Methods:**

In previous work we have developed a model predictive control (MPC) based method for determining optimal treatment interruption schedules for this purpose. In this paper, we introduce a nonlinear observer for the HIV-immune response system and an integrated output-feedback MPC approach for implementing the treatment interruption scheduling algorithm using the easily available viral load measurements. We use Monte-Carlo approaches to test robustness of the algorithm.

**Results:**

The nonlinear observer shows robust state tracking while preserving state positivity both for continuous and discrete measurements. The integrated output-feedback MPC algorithm stabilizes the desired steady-state. Monte-Carlo testing shows significant robustness to modeling error, with 90% success rates in stabilizing the desired steady-state with 15% variance from nominal on all model parameters.

**Conclusions:**

The possibility of enhancing immune responsiveness to HIV through dynamic scheduling of treatment is exciting. Output-feedback Model Predictive Control is uniquely well-suited to solutions of these types of problems. The unique constraints of state positivity and very slow sampling are addressable by using a special-purpose nonlinear state estimator, as described in this paper. This shows the possibility of using output-feedback MPC-based algorithms for this purpose.

## Background

The majority of untreated HIV patients, following a brief period of acute infection, enter a long asymptomatic phase of infection characterized by high viral loads, persistent immune activation, and a slow decline in the helper-T cell concentration [1]. Eventually, the concentration of helper-T cells becomes too low to sustain effective immune responses, and opportunistic infections cause a dramatic decline in the patient's health. The slow decline of helper-T cells during the asymptomatic phase was once thought to indicate a slow rate of infection and cell turnover, but it is now known that very fast rates of virus and host cell turnover, as high as 10^10 ^virions per day or 2 × 10^9 ^infected helper-T cells per day occur during this phase [2,3].

The majority of patients follow the disease progression pattern described above, but a small number of untreated patients, termed Long-Term Non-Progressors, do not show progressive decline in helper-T cell counts, have consistently low measured viral loads, and do not show impaired immune responses, and show strong HIV-specific helper-T cell responses [4,5]. Levels of Cytotoxic-T cells specific to HIV in these patients remain high, even at low viral loads [6,7]. Patients with progressive HIV infection show a dramatic drop in the level of these cells when the viral load is reduced [8,9]. Long-Term non-progressors can transition to progressive HIV infection [10], probably due to the evolution of HIV strains resistant to the immune response [11].

In order to prevent mutation escape of the virus, HIV therapy uses three antiviral drugs simultaneously. These drugs, which target different epitopes in the HIV genome, make it very unlikely that the virus can simultaneously evolve resistance to all three drugs. This approach, called Highly Active Anti-Retroviral Therapy (HAART) is very effective at reducing viral load [12]. Unfortunately, the drugs used in HAART have a number of significant adverse side effects, and must be continued for the life of the patient [13]. HAART interruptions have been investigated in order to manage side effects of treatment or to allow treatment of secondary infections such as hepatitis-A [14-16]. A small number of cases where therapy was started during acute infection and then discontinued and re-initiated have apparently led to long-term, drug-free suppression of the virus [17,18]. Follow-up studies investigating structured treatment interruptions (STI) as a method of inducing immune-mediated control of the virus showed some success in inducing a transient immune-mediated control of the virus [19-23]. Patients showing viral control also showed increased HIV-specific helper-T cell counts and increased HIV-specific cytotoxic-T cell counts, similar to the pattern seen in LTNPs. Follow-up studies tracking these patients showed that a majority of these patients eventually reverted to an actively progressing infection [24].

Studies of STI in patients who originally initiated treatment during chronic infection showed no success in inducing immune-mediated control, suggesting that treatment initiation during acute infection is a necessary condition for success in this approach [25-33]. HIV is known to preferentially infect HIV-specific T-cells [34], so HIV-specific helper-T cell pools may be permanently damaged in patients that delay therapy until the chronic phase of infection [35-39].

The use of STIs in HIV therapy is controversial [40]. Interruptions in therapy are likely to encourage the evolution of drug-resistance mutations [41-43]. It is clear that before these STI-based methods will be attempted again, a reliable model of resistance risk will need to be developed. This is the focus of much of our recent research [44-47]. Although STI-induced immune control has shown disappointing durability on its own, it could still be used in conjunction with a reduced-dosage HAART to attain similar levels of viral suppression with fewer side effects. Assuming that the immune response affects different targets from the HAART, this regimen should also be more durable than HAART alone. Some evidence exists for the possibility of durable immune control, as reported in [48]. Nevertheless, it will be necessary to increase the success rate of STI in inducing immune-mediated control, and find methods of moderating the risk of resistance evolution, for this method to become a viable option for HIV therapy.

In previous work, we developed a Model Predictive Control (MPC) based method for finding these schedules. This method is well-suited to the problem for a number of reasons: It is easily adaptable, which will allow for various improved models to be integrated as they are developed. It inherits from the MPC framework a certain robustness to disturbances and model inaccuracies which is important, since the model in question is known to suffer from these. It allows us to fine-tune the treatment using medically intuitive notions of cost. Finally, the long time-scales of the model allow us to overcome the computation time issues which normally plague MPC-based methods. However, the original work in [49] assumed full-state measurements. In practice only viral load measurements can be made with the frequency and accuracy necessary for a feedback control method.

In this paper, we introduce a full nonlinear observer with acceptable properties, and test its reliability in the face of model uncertainty. This serves as a "proof of concept" study for the use of nonlinear-observer output-feedback MPC in treatment scheduling for HIV. Other authors have also considered similar problems. The authors of [50] introduced an output-feedback model predictive control-based method for treatment scheduling for a different but related model of HIV dynamics, using an Extended Kalman Filter as the observer. The performance of this estimator began to rapidly degrade with model parameter uncertainty; however, a one-to-one comparison is not possible as the model of HIV dynamics was not the same. The authors of [51,52] also developed an output feedback MPC algorithm for treatment scheduling of a different model of HIV infection; this paper used a deadbeat observer and assumed the ability to measure both CD4^+ ^T-cell count and viral load, instead of just viral load as in this paper. The authors of [53] considered open-loop finite-horizon optimal control of a very simple model of HIV infection, allowed continuous varying of drug concentration, and did not consider either the measurement problem or the model inaccuracy problem. The authors of [54] introduced a robust multirate MPC controller to calculate treatment schedules for a model of HIV infection that does not include immune response dynamics, allowing continuous variation of drug dosing. The authors of [55,56] developed an innovative output feedback scheduling method for the same model which we use, but assume that both the CD4^+ ^T-cells and viral load are measurable. Their method does not use an MPC scheduling method. The authors of [57] present an output-feedback method for controlling a variation of the model which we use; however, their approach allows for continuous values of drug dosing, unlike our method which assumes constant drug dosing of either 1 or 0. The authors of [58] introduced a sophisticated nonlinear observer design for the same HIV model used in this work, with very good convergence in the continuous measurement case. Their method, however, relied on direct estimates of higher-order derivatives, which required sampling every 6 hours during the early phase of treatment, compared with the 1-week intervals proposed in this work. The authors of [59] consider the treatment scheduling problem as a multi-objective optimization and obtain a Pareto frontier, incidentally showing the near-optimality of our previously reported results in [49]. The authors of this paper do not consider the problem of output feedback. A review of the various control approaches applied to HIV medicine was presented in [60].

The paper is organized as follows. We first introduce the Wodarz-Nowak model of HIV infection. Next we introduce the nonlinear observer design. We then show the performance of this observer with continuous measurements and sampled measurements. Next we introduce the complete output-feedback MPC-based treatment scheduling method, which combines the full-state feedback MPC of [49,61] with the observer introduced in the following section. Finally, we evaluate the performance of this design through Monte-Carlo experiments with various levels of model uncertainty. The conclusions discuss the implications of these results for future work in this area. This paper is the first to present a nonlinear-observer based output-feedback MPC algorithm for HIV treatment scheduling that incorporates realistic constraints on measurement intervals and relies only on the highly accurate viral load measurements.

## Results and Discussion

### Model

We consider a five-state nonlinear ODE model of HIV infection and immune response introduced in [62].(1)

where **x **represents the concentration of healthy helper-T cells, **y **represents the concentration of HIV-infected helper-T cells, **z**_1 _represents the concentration of inflammation mediated cytotoxic-T cells, **w **represents the concentration of memory phenotype cytotoxic-T cells, and **z**_2 _represents the concentration of helper-T cell mediated cytotoxic-T cells. **u **is a binary variable representing the application of HAART, and *η *is HAART's effectiveness at reducing the infection rate. All states lie in the non-negative orthant, which is also positive invariant. **u**(*t*) is restricted to take values of either 0 (no treatment) or 1 (full treatment), in order to avoid the rapid evolution of the virus likely under suboptimally suppressed conditions. The measurable output, plasma viral load, is proportional to the infected cell state **y**, due to a singular perturbation phenomenon (the decay rate of the free virus is much faster than the death rate of infected cells). A more complete description of the states and their interactions can be found in our previous paper [49].

For realistic parameter values in the absence of treatment, this model has two stationary points in the non-negative orthant. One of these corresponds to a state where the virus is controlled by the immune response:(2)

and one in which the virus dominates, progressing to AIDS:(3)

This model in this paper uses normalized parameter values.

### Observer Design

This application presents some unique challenges for observer design. The system described by Equation 1 is nonlinear with multiple steady-states. Observer design for such a system is very much an open problem. Also, the invasive nature of blood-drawing methods puts a very coarse lower limit on sampling time, with intersample times of one week a bare minimum. The observer must therefore be reasonably robust to error due to sampling.

After experimenting with simple high-gain type observers, we discovered they performed poorly in a sampled-data situation. The bare error injection term would, for large enough initial error, cause the observer to violate the non-negative orthant restriction of the original system, which wreaked havoc on the numerical simulator as well as being completely unrealistic. For the purpose of this paper, we settled on a nonlinear observer specific ally designed to satisfy the non-negative orthant restriction. The observer design was heavily influenced by the symmetry-preserving observer concept presented in [63], though we did not follow the same formal design approach. By starting with a copy of the system, and allowing output error-correction terms to enter the system in a manner following the system's natural geometry, we obtained an observer design that preserved state positivity, ensured good convergence, and avoided unrealistic patterns of intermediate state estimate values while the system was converging. The equations describing this observer are(4)

where **x_e_**, **y_e_**, **z_1e_**, **w_e_**, **z_2e _**are the state estimates of **x**, **y**, **z_1_**, **w**, **z_2 _**respectively. We let **X **refer to the vector of all states and **X_e _**refer to the vector of state estimates. This yields error dynamics described by the equations(5)

where **e_X _**is **X - X_e_**. The combined system of Equations 1 and 5 have two steady-state solutions, corresponding to the steady-state values for the original system described in Equations 2 and 3 combined with the error values **e_X _**= 0. For the parameter values of Table 1, these two steady-states are locally stable by Lyapunov's second method.

**Table 1:** Parameter Values

Parameter	*d*	*β*	*a*	*p*_1_	*p*_2_	*c*_1_	*c*_2_	*b*_1_	*b*_2_
Value	0.1	1	0.2	1	1	0.03	0.06	0.1	0.01
**Parameter**	**λ**	***q***	**η**	***^K^*1**	***^K^*2**	***^K^*3**	***^K^*4**	***^K^*5**	
Value	1	0.5	0.9799	10	10	150	5	50	

These are the parameter values used in our implementation of the nonlinear observer, values adapted from [62].

### Simulations

**Figure 1:**
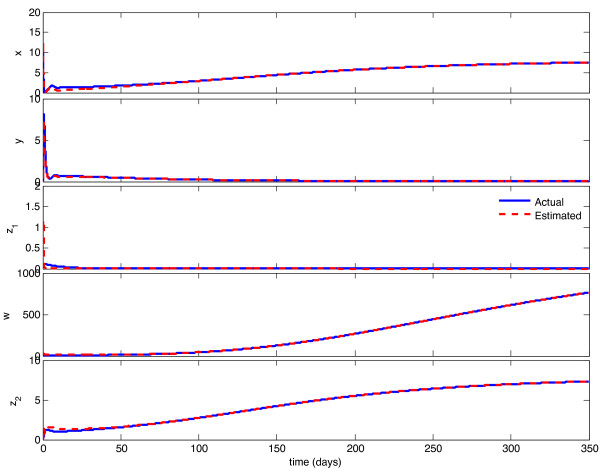
**Continuous-time observer convergence - same ROC**. Initial estimate and initial condition are in the same Region of Attraction. Initial state is {**x **= 10, **y **= 0:1, **z_1 _**= 0.1, **w **= 3, **z_2 _**= 0.1}, initial estimate is {**x_e _**= 10, **y_e _**= 3, **z_1e _**= 1, **w_e _**= 10, **z_2e _**= 0:1}.

**Figure 2:**
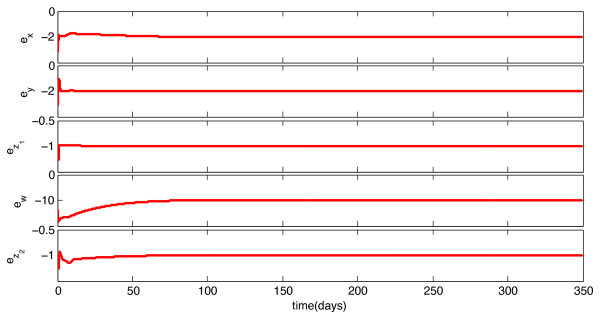
**Continuous-Time Observer Error Convergence**. Initial Estimate and Initial Condition are in the same Region of Attraction.

**Figure 3:**
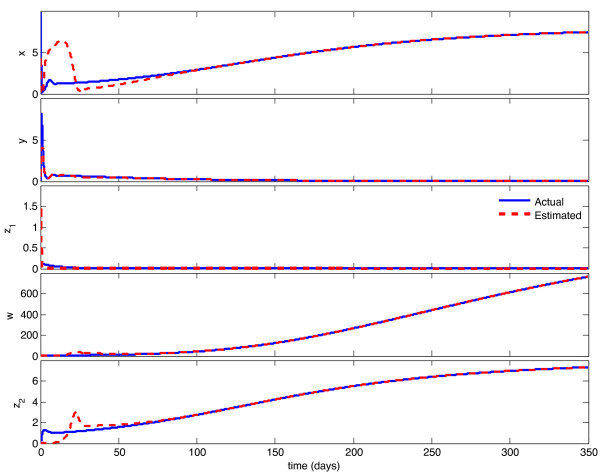
**Continuous-time observer convergence - different ROC**. Initial estimate and initial condition are in different Regions of Attraction. Initial state is {**x **= 10, **y **= 0.1, **z_1 _**= 0.1, **w **= 3, **z_2 _**= 0:1}, initial estimate is {**x_e _**= 2, **y_e _**= 3, **z_1e _**= 1, **w_e _**= 0.1, **z_2e _**= 0.1}.

**Figure 4:**
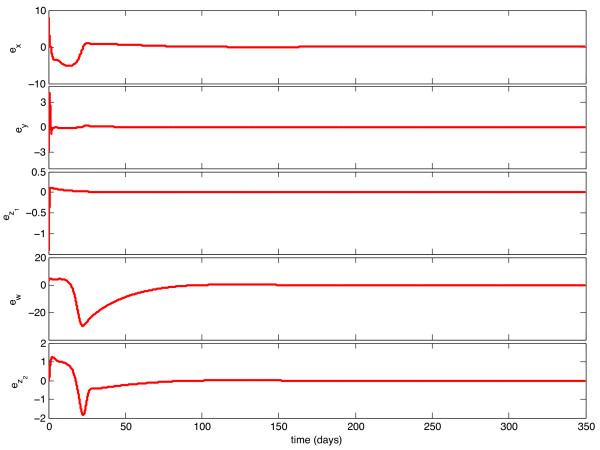
**Continuous-Time Observer Error Convergence**. Initial Estimate and Initial Condition are in different Regions of Attraction.

We implemented the observer described above, with model parameters as listed in Table 1, and tested its behavior under a variety of circumstances. With continuous feedback, the observer error converged asymptotically to zero for every combination of initial state and estimate. Representative examples of this can be seen in Figures 1 and 3. In Figure 1, the initial condition is in the same Region of Attraction (ROA) as the initial estimate (error convergence for this case is shown in Figure 2), and in Figure 3, the initial estimate is in a different ROA from the initial condition (error convergence for this case is shown in Figure 4). The location of initial conditions in a particular ROA was verified by simulation. In both cases the estimate converges to the actual value.

**Figure 5:**
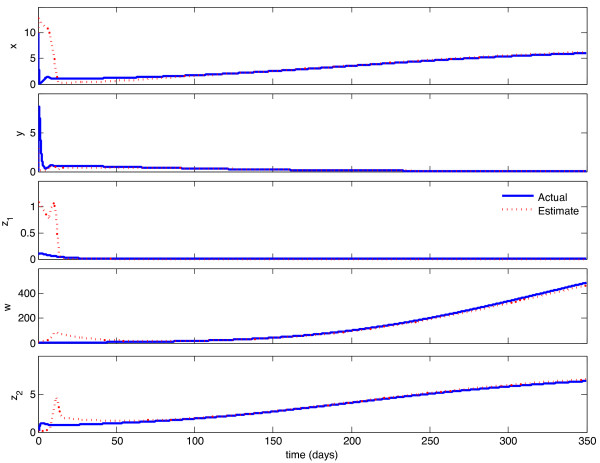
**Discretized observer convergence - same ROC**. Initial estimate and initial condition are in the same Region of Attraction. Initial state is {**x **= 10, **y **= 0:1, **z_1 _**= 0.1, **w **= 3, **z_2 _**= 0.1}, initial estimate is {**x_e _**= 10, **y_e _**= 3, **z_1e _**= 1, **w_e _**= 10, **z_2e _**= 0.1}.

**Figure 6:**
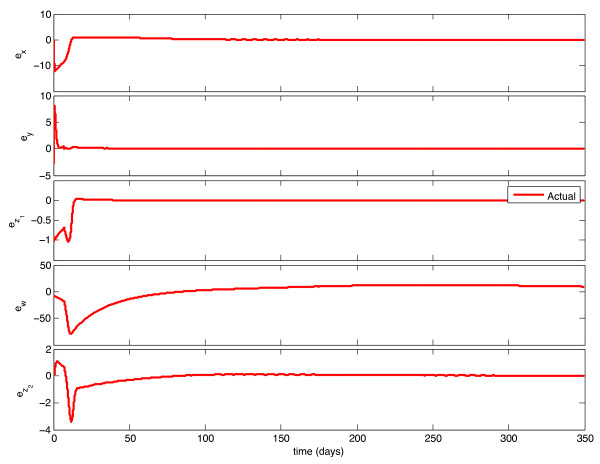
**Discrete-Time Observer Error Convergence**. Initial Estimate and Initial Condition are in the same Region of Attraction.

**Figure 7:**
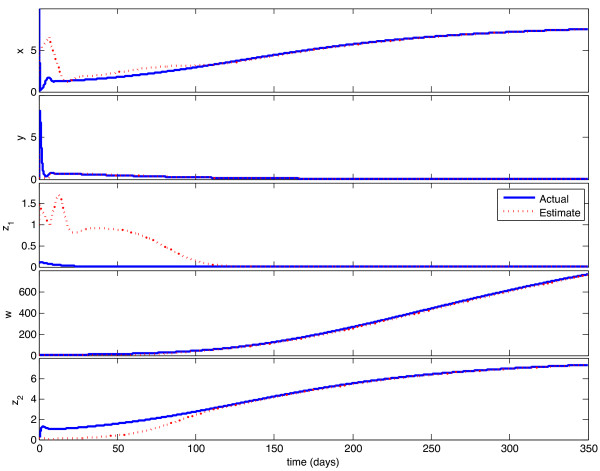
**Discretized observer convergence - different ROC**. Initial estimate and initial condition are in different Regions of Attraction. Initial state is {**x **= 10, **y **= 0.1, **z_1 _**= 0.1, **w **= 3, **z_2 _**= 0.1}, initial estimate is {**x_e _**= 2, **y_e _**= 3, **z_1e _**= 1, **w_e _**= 0.1, **z_2e _**= 0.1}.

**Figure 8:**
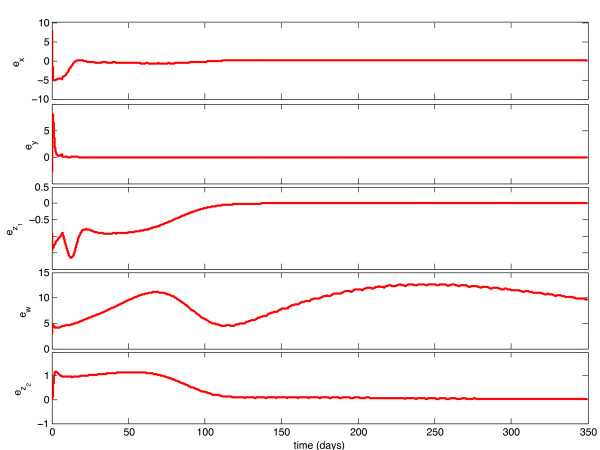
**Discrete-Time Observer Error Convergence**. Initial Estimate and Initial Condition are in different Regions of Attraction.

The actual system is constrained by a sampling period of no less than one week. Accordingly, we implemented in MATLAB a discretized sample and hold version of the continuous observer described above, with a sampling period of one week. Again, this observer performed well, with error converging toward zero, albeit at a slower rate than in the continuous-time case. Figure 5 shows the performance of the discretized observer when the initial condition is in the same ROA as the initial estimate (error convergence for this case is shown in Figure 6), and Figure 7 shows the performance when the initial condition is in a different ROA than the initial estimate (error convergence for this case is shown in Figure 8). It should be noted that the tuning parameters (*K *_1_, *K *_2_, *K *_3_, *K *_4_, *K*_5_) used in these simulations are tuned for performance of the discretized observers. Better performance is possible for the continuous-time observer, at the cost of higher gains and worse performance for the discretized observer. The parameters (*K *_1_, *K *_2_, *K *_3_, *K *_4_, *K *_5_) were tuned primarily through trial and error; local stability of the observer holds for a wide range of values, but there is a clear trade-off between convergence rate and sensitivity to low sampling rates.

### Output Feedback

In this section, we present the output feedback adaptation of the Model Predictive Control based treatment scheduling algorithm of [49]. Output-feedback Model Predictive Control recursively solves a finite-horizon optimal control problem, applying the first sample of each optimal control solution before resampling the system. Consider a discrete-time system of the form(6)

and a current measurable output **y_k _**= g(**X_k_**) we find a length N sequence **U **= {**u_k_**, **u**_**k**+**1**_, ..., **u**_**k**+**N-1**_} which minimizes a cost function of the form(7)

with stage cost *l*, terminal cost *F*, and state estimate **X_e _**obtained from the observer. The first element of the resulting optimal control sequence is applied, a new sample is obtained, and a new optimal control sequence is calculated. An excellent review of MPC in its various implementations can be found in [64]. Our control objective is to globally stabilize the stationary point described in Equation 2. We also want to minimize the transient decrease in helper-T cell concentration. We use the stage cost:(8)

where *α_j _*> 0 are design weights and *x_LTNP _*, *w_LTNP _*are the desired equilibrium values for the respective states [65]. shows conditions on the full-state feedback system and controller which, if satisfied, guarantee robust asymptotic convergence to a neighborhood of the desired equilibrium. In a similar fashion, the work in [66] shows conditions on the system, output, observer, and state-feedback MPC formulation which, if satisfied, allow the use of the state-feedback MPC algorithm with the estimated state values from the observer to generate an output-feedback MPC algorithm which robustly stabilizes the desired steady-state. We implemented the output-feedback MPC algorithm described above in MATLAB. With no error in the model parameters, across a large range of randomly selected initial conditions and initial estimates, the algorithm always managed to stabilize a small neighborhood of the desired steady-state of Equation 2.

#### Robustness

**Figure 9:**
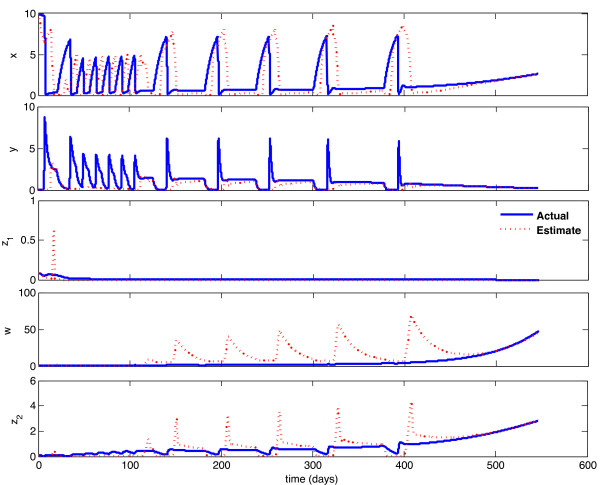
**Output Feedback MPC robust performance**. Initial estimate and initial condition are the same, controller uses nominal parameters as in Table 1, parameters in the model vary randomly from these by as much as 10%.

While the measurements of viral load (proportional to output **y**) have well-characterized log-normal variation, the parameters of the system are expected to vary significantly from patient to patient, and are impossible to identify in practice for each patient. In [49], we characterized the robustness of the state-feedback system to error in the model. We introduced a at random variation into every parameter in the model. The scheduling algorithm continued to use the nominal, but now incorrect values to calculate its schedules. We ran at least 100 simulations each with this error randomly distributed at up to 5%, 10%, 15%, 20%, 25%, and 30% of each parameter value, allowing the algorithm up to two (patient) years to successfully stabilize the desired steady-state. The simulations were carried out from a common initial condition. We run the same Monte-Carlo type robustness test here on the output-feedback MPC algorithm described in this section, and the results are summarized in Table 2 with the results from [49] included for comparison. An example of typical performance is shown in Figure 9. As expected, the output-feedback algorithm was outperformed by the state-feedback algorithm, but the success rate of the output-feedback algorithm did not drop o dramatically as model error increased, and even at up to 30% error in every parameter, is still better than 70%. The inconsistencies between the 25% and 30% error cases are undoubtedly due to the small sample sizes, which in turn were forced by the computational cost of these simulations. These results demonstrate a practical robustness to modeling error which strongly motivates the use of output-feedback MPC in treatment scheduling for HIV.

**Table 2:** Robustness to modeling error

	State-Feedback MPC		Output-Feedback MPC	
% Error	Success Rate	# of samples	Success Rate	# of trials
5%	100%	100	100	107
10%	100%	100	98.1	106
15%	100%	115	90.2	102
20%	99.4%	140	81.9	105
25%	98%	100	71	107
30%	90.7%	129	72.5	102

This table compares the degree of flat-random error in the parameter estimates with the success rate of the feedback algorithm in stabilizing the desired outcome.

## Conclusions

In this paper, we have introduced a candidate nonlinear observer for use in output-feedback MPC-based treatment scheduling for HIV. The observer is designed to preserve the forward-invariance of the non-negative orthant in the face of sampling-induced measurement error. The observer performs well in both the continuous-time and discretized implementations.

We have implemented an output-feedback MPC-based scheduling algorithm, and tested its robustness to modeling error. The closed-loop system performed well. Also, the performance of this output-feedback system should be understood as a lower-bound on what is possible. This work motivates the use of output-feedback MPC, but the observer used is only one candidate observer. A more natural implementation might be a nonlinear receding-horizon observer as in [67], though the implementation of such an observer for a system such as ours is still an open problem.

The possibility of enhancing immune responsiveness to HIV through dynamic scheduling of treatment is exciting. Model Predictive Control is uniquely well-suited to solutions of these types of problems. The sample-and-prescribe framework is reconcilable to the realities of patient treatment through office visits. The work in this paper shows the possibility of using output-feedback MPC-based algorithms for this purpose.

## Competing interests

The authors declare that they have no competing interests.

## Authors' contributions

RZ conceived of the study, designed all methods, implemented all methods, and drafted the manuscript.
